# Aseptic Loosening of Smaller Corail Stems in Heavy, Active Males: A Report of Three Cases

**DOI:** 10.7759/cureus.14563

**Published:** 2021-04-19

**Authors:** Andrew G Yun, Marilena Qutami, Eric Carles

**Affiliations:** 1 Orthopaedic Surgery, Center for Hip and Knee Replacement, Providence Saint John's Health Center, Santa Monica, USA; 2 Center for Hip and Knee Replacement/Orthopaedic Surgery, Providence Saint John's Health Center, Santa Monica, USA

**Keywords:** failed total hip, aseptic loosening, corail stem

## Abstract

Recent concerns have been raised regarding a higher failure rate with smaller size Corail stems. This case series examines early aseptic loosening with smaller stems in three large male patients with Dorr A bone. Each stem was fluoroscopically aligned and sized until stable with axial and rotational stress. In each case, failure occurred within six months due to symptomatic metaphyseal debonding. Careful analysis suggests a correlation of failure to small size stems that are comparatively 1) undersized relative to the metaphysis, 2) undersized relative to patient body mass index, and 3) undersized relative to the amount of offset created.

## Introduction

The Corail stem is the most widely used femoral implant in the Australian, Norwegian, and National Joint Registries (NJRs) [1-4]. Long-term results are excellent, with reported survival rates at 96.3% at 23 years [5]. The Orthopedic Data Evaluation Panel awarded the stem its highest 10A rating [6]. In the 2020 American Joint Replacement Registry, the Corail is the second most commonly used cementless stem and has the highest percentage free from revision (99.1% of 26,714 Corail stems) among the most commonly used stems [7].

Despite the outstanding long-term success, careful registry analysis of the Corail has identified an increased risk of aseptic loosening in certain stem sizes. The standard stem sizes range from 8 to 20, with an incremental increase in stem width, cross-sectional area, and length. In a retrospective study of 35,386 hips with the Corail stem from the NJR of the United Kingdom, Jameson et al. observed a statistically significant higher risk of failure with smaller stems of sizes 8-10 [8]. Similarly, in a review of 41,265 Corail stems in the Australian Joint Replacement Registry, Hoskins et al. also noted a significantly higher cumulative percent revision rate in smaller stems sized 8-9, a rate that rose even higher with a direct anterior (DA) approach [9]. These large database registry studies, however, could not explain these findings [8].

Although we too have found the Corail to be a highly successful stem, we have encountered three instances of early failure due to aseptic loosening despite appropriate sizing and alignment. Each occurred within the first six months in patients with the same three risk factors for revision identified in the aforementioned registry studies: heavy, active men with small Corail stems. The purpose of this case review is to correlate the macroscopic database findings with the radiographic progression of early Corail stem failure. 

## Case presentation

Methods

From 2010 to 2019, a total of 5,838 primary DA approach total hip arthroplasties (THAs) with Corail stems were performed by a single surgeon using a Hana table and fluoroscopy. All cases were performed following standard Corail technique guidelines. We retrospectively reviewed three patients who developed early aseptic loosening due to metaphyseal debonding of the femoral stem. At the index procedure, the stems were placed in neutral alignment with fluoroscopy confirming both position and sizing. Surgery was performed at a single institution with a minimum of two-year follow-up. Patients who underwent revision in this series had failed conservative treatment and demonstrated signs of radiographic loosening with varus migration and progressive radiolucent lines (RLLs) in the proximal stem. Patients requiring stem revision due to sepsis or periprosthetic fracture were not included. Medical records were reviewed for indications, demographics, clinical history, and implant sizes at the time of primary surgery. Secondarily, records were reviewed for complications, reoperations, and readmissions. The study was approved by the Institutional Review Board.

Surgical technique

**Figure 1:**
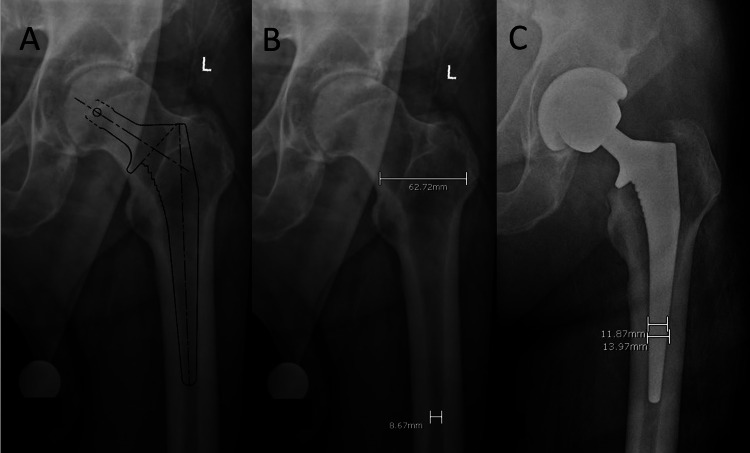
Radiographic examples of templating and measurements. (A) Calibrated template for stem size, hip length, and offset. (B) Sample measurements used to calculate Noble canal-flare index. (C) Sample measurements used to calculate stem-to-canal fill percentage.

The patient was placed on the Hana table in the standard fashion. A DA approach was performed as described by Matta et al. [10]. Preoperative and intraoperative templating with calibrated images were used to estimate sizing, stem type, and head length needed to restore hip length and offset (Figure 1A). A standard-length, extensively coated hydroxyapatite design was used consecutively in all primary cases using a broach-only technique as recommended [11]. The cementless stem is a titanium alloy with a trapezoidal proximal body for metaphyseal fixation and a tapered quadrangular distal taper to avoid distal diaphyseal blocking. A collared design was always used in KA standard and KLA coxa vara stems, and a collarless design was used in the early KHO high-offset design since no collar was available at the time. Final stem sizing was based on 1) axial and rotational stability of the final trial broach, 2) radiographic fill and alignment, and 3) confirmation of sizing with preoperative templating. With the trials in place, intraoperative fluoroscopy was used to confirm implant sizing and alignment in the canal, as well as hip length and offset. Trial stems that were noted to be in a varus position fluoroscopically were repositioned into a neutral coronal alignment after proximal lateralization. Clinically, trial stems that demonstrated motion with rotational stress were either impacted more deeply or upsized until rotationally stable. The trials were tested for stability and impingement through a range of motion before the final implants were placed.

In this series, patients were allowed to progress with weight bearing as tolerated and without dislocation precautions. Patients were anticoagulated with aspirin for three weeks unless stronger anticoagulation was specifically indicated based on risk stratification, degree of immobility, and prior medical history.

Radiographic measurement

Preoperative radiographs were evaluated for femoral bone morphology based on the Dorr classification (A, B, and C) using the canal-calcar ratio and the Noble classification (champagne-flute, normal, stovepipe) using the canal-flare index (Figure 1B) [12,13]. Postoperative radiographs of the stem were evaluated for alignment in two planes and stem-to-canal fill percentage (Figure 1C) [14]. On follow-up imaging, implants were evaluated for loosening, migration, and RLLs as previously well described [15].

Case 1

**Figure 2:**
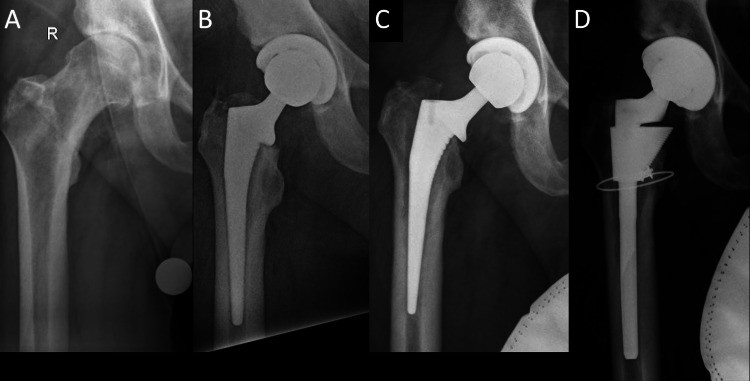
Radiographs of patient 1. (A) Preoperative AP of patient 1 with Dorr A bone and canal-flare index of 5.8. (B) Postoperative AP of Corail size 10, collared, high-offset stem with a +5 mm head. (C) Follow-up radiographs with radiolucent lines in zones 1 and 7 with varus migration. (D) Revision of S-ROM stem. AP, anteroposterior view

Patient 1 is an active 59-year-old man with a body mass index (BMI) of 34. His femur showed Dorr A bone type and a high canal-flare index of 5.8 (Figure 2A). He underwent uncomplicated primary DA THA with a size 10 Corail collared stem with a high-offset neck and a 36 mm +5 mm head. Postoperative radiographs showed neutral coronal and sagittal alignment of the stem and a canal fill ratio of 0.91 (Figure 2B). He progressed rapidly in the early recovery period. He was walking 5 miles a day and back to full-time work four weeks following surgery. At eight weeks postoperatively, he reported new symptoms of thigh pain, swelling, and weakness. Radiographs showed development of 3-5 mm thick RLLs in zone 1 coronally and zones 8 and 14 laterally (Figure 2C). Despite conservative measures including a cane and activity restriction, his symptoms continued to escalate, and he was ultimately revised to an S-ROM 16 x 11 x 36 + 6 stem through a posterior approach (Figure 2D). The modular fixed bearing liner was revised to a dual-mobility construct for enhanced stability. Postoperatively, the patient was placed on restricted weight bearing with limited activity. He returned to work part time after four weeks and resumed full weight bearing and full-time work eight weeks following revision.

**Figure 3:**
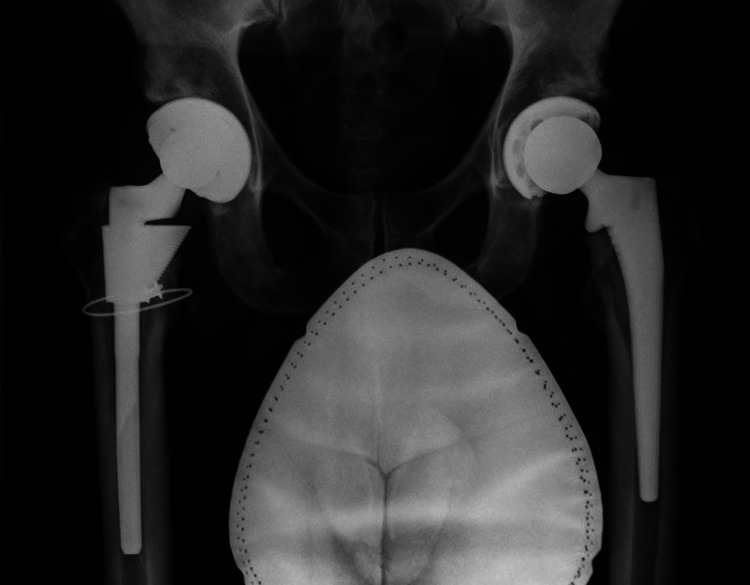
Two-year follow-up showing right hip revision and left hip replacement with a Corail size 12, collared, coxa vara stem and a +1.5 mm head.

Of note, this patient recovered well and returned to our office for treatment of advanced arthritis in his opposite hip. After careful consideration of stem options and surgical approaches, we proceeded with a Corail stem through a DA approach. However, for this hip we reamed the distal canal in an effort to get improved metaphyseal fixation with a larger stem. By widening the diaphysis, we were able to increase the stem size from a 10 to a 12. A KLA collared stem with a +1.5 head was used to restore leg length and offset (Figure 3). Postoperatively, the patient was allowed to progress to full weight bearing with activity restriction. He was back to work full time without activity restriction at eight weeks. At one year following surgery, the patient continues to do well. 

Case 2

**Figure 4:**
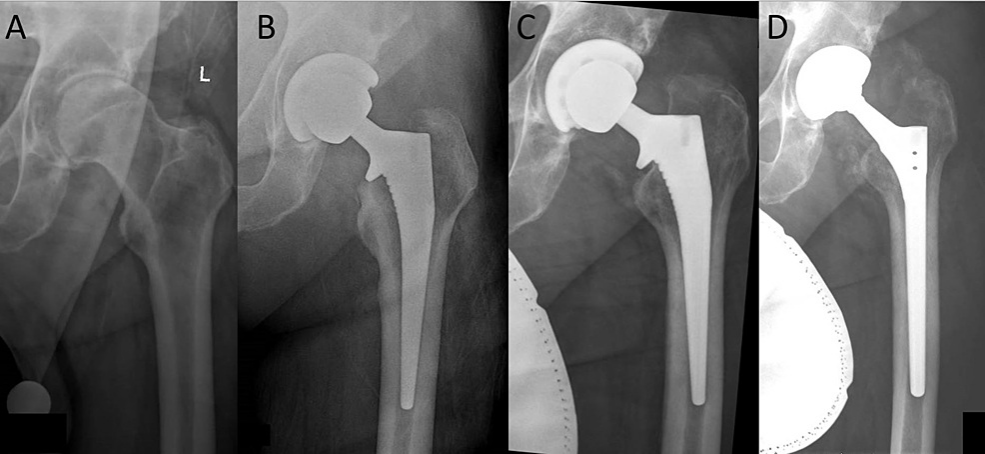
Radiographs of patient 2. (A) Preoperative AP of patient 2 with Dorr A bone and canal-flare index of 7.8. (B) Postoperative AP of Corail size 11, collared, coxa vara stem with a +5 mm head. (C) Follow-up radiographs with varus migration. (D) Revision with Wagner monoblock stem. AP, anteroposterior view

Patient 2 is a 65-year-old man with a BMI of 37. His femur showed Dorr A bone type and a high canal-flare index of 7.8 (Figure 4A). He underwent uncomplicated primary DA THA with a Corail size 11 coxa vara collared stem and a 36 mm +5 mm head. Postoperative radiographs showed neutral coronal and sagittal alignment of the stem and a canal fill ratio of 0.88 (Figure 4B). The patient did well for the first few weeks following surgery. Five weeks postoperatively, he described an increase in pain. He reported that he was only able to walk 5 minutes and required a cane and pain medication. Conservative measures including activity restriction, ice, and elevation were unsuccessful. Radiographs revealed a new development of RLLs in zones 1, 7, 8, and 14 with varus migration (Figure 4C). The patient underwent revision surgery through a posterior approach using a monoblock Wagner 15 x 190 stem (Figure 4D). The modular fixed bearing liner was revised to a dual-mobility construct for enhanced stability. Postoperatively, the patient was put on restricted weight bearing for 12 weeks. He was back to work with unrestricted activity at final follow-up.

Case 3

**Figure 5:**
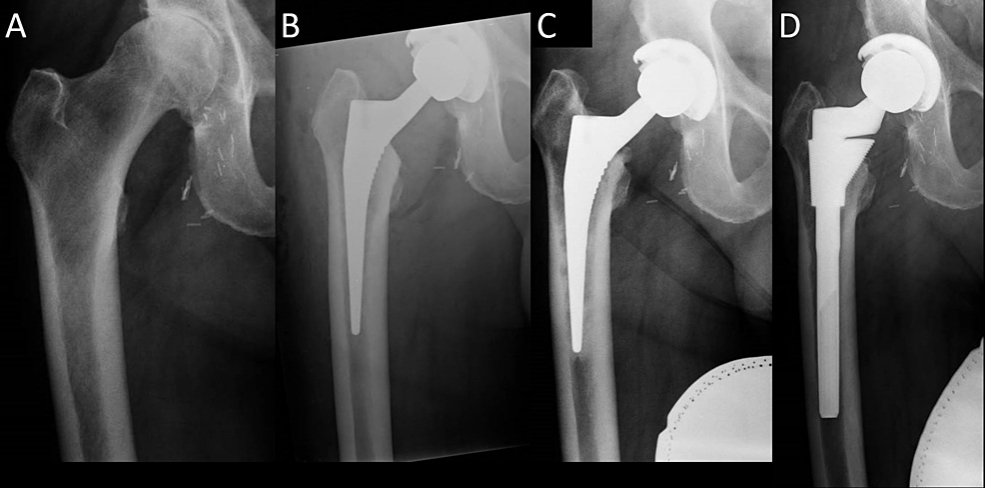
Radiographs of patient 3. (A) Preoperative AP of patient 3 with Dorr A bone and canal-flare index of 4.9. (B) Postoperative AP of Corail size 9, collared, high-offset stem with a +5.5 mm head. (C) Follow-up radiographs with subsidence and varus migration. (D) Revision of S-ROM stem. AP, anteroposterior view

Patient 3 is a 61-year-old man with a BMI of 30. His femur showed Dorr A bone type and a high canal-flare index of 4.9 (Figure 5A). He underwent uncomplicated DA THA with a size 9 Corail collarless stem with a high-offset neck and a 32 mm +8.5 mm head. Postoperative radiographs showed neutral coronal and sagittal alignment of the stem and a canal fill ratio of 0.81 (Figure 5B). The patient recovered well and quickly progressed to walking 2 miles daily. He reported new-onset start-up pain within five months with an inability to walk long distances secondary to thigh pain. Radiographs showed lack of metaphyseal ingrowth with varus migration and subsidence (Figure 5C). A bone scan was ordered, which showed increased uptake consistent with loosening. Revision was offered to the patient, but he preferred to defer surgery hoping that the stem might stabilize. He presented to the office five months later with 9/10 thigh pain. The patient underwent stem revision through a posterior approach using an S-ROM 18 x 13 x 36 standard stem (Figure 5D). Postoperatively, the patient was put on restricted weight bearing with a walker for three weeks followed by full weight bearing with an assistive device for balance for an additional four weeks. Eight weeks following revision surgery the patient reported feeling well and walking comfortably. He continued to progress well and eventually returned to our practice for evaluation and treatment of a different arthritic joint.

## Discussion

Aseptic loosening in the Corail stem is a relatively rare event. The concerns raised by Jameson et al. and Hoskins et al. prompted us to examine our own failures more carefully in a search for patterns [9]. In our experience, failure as described by Jameson et al. from aseptic loosening of the stem occurred in only three of 5,838 hips (0.05%) [8].We recognize that other patients with aseptic stem failure may have left our practice to seek treatment elsewhere and that the real rate of failure may be higher. Nonetheless, we believe our experience to be consistent with the excellent stem survival rates found in all major joint registries.

The etiology of failure in these smaller stems, however, is unclear. Using a database without specific patient information, Jameson hypothesized that failure could be secondary to an “inadequate fit or poor bone quality” [8]. In this series, however, these small stems failed despite an adequate fit and overall excellent bone quality. Another potential variable contributing to early femoral loosening is the use of a DA approach. A possible concern is that the DA approach may contribute to placement of the stem in varus. Using a DA approach, the incidence of Corail stem malalignment in a varus position was noted to be 59% by Haversath et al. [16] and 40% by Batailler et al. [17]. In our series, however, intraoperative fluoroscopy was used to confirm a neutral alignment of the trial within the canal before stem insertion. Thus, it appears that other factors are likely to contribute to small stem size failure.

The Corail is ideally designed for metaphyseal fixation, and these three failures exhibited failure to ingrow at the metaphysis. The time to failure was short with these patients complaining of worsening pain within the first five months. Follow-up radiographs demonstrated progression of proximal RLLs with progressive varus migration. This radiographic pattern of failure suggests that cantilever forces may play a role. Careful analysis of these cases suggests a correlation of failure with small size stems that are comparatively 1) undersized relative to the metaphysis, 2) undersized relative to patient BMI, and 3) undersized relative to the amount of offset needed to match the opposite hip.

Undersizing relative to the metaphysis may occur when a narrow distal canal blocks entry of a larger stem. The distal taper catches in the diaphysis and prevents further broach impaction. The femoral bone in these three cases demonstrated a preoperative high diaphyseal-metaphyseal mismatch. All three femurs had an average canal-flare index of 6.2 and were Dorr A bone with a thick diaphyseal cortex distally. Similarly, Magill et al. highlight the importance of templating to recognize a potential mismatch to avoid the use of small stems when possible, especially in Dorr A bone [16]. Early locking of the distal taper of the broach into a narrow diaphysis limits entry of larger sizes.

Currently, in cases where a small stem is templated due to the narrow distal canal of Dorr A bone and a canal-flare index greater than 4.8, we routinely use reamers to widen the distal canal as described in the Corail technique guide. With an anterior approach, straight reamers are difficult to angle properly; however, flexible reamers over a guidewire can safely expand the distal canal with minimal risk of perforation. The canal can then often be broached one to two additional sizes for an improved metaphyseal fill, as demonstrated in patient 1. To date, we are not aware of any clinical studies on the results of the reaming technique, although the manufacturer’s technique guide also suggests reaming if the stem is binding distally. In heavy, active males with Dorr A bone where a larger-size stem (size 11 or above) is not achievable, an alternative stem design is used.

The stems in our case reports may also have been undersized in relation to body size. In our three cases, failure occurred in patients with a mean weight of 254 and a mean BMI of 33. With the Corail, Buttaro identified a failure risk higher in overweight patients [18], and Al-Najjim also noted a correlation of Corail subsidence with increased BMI and more active patients [19]. The larger forces of high bodyweight may have placed too much angular stress on the more narrow stem geometry in the smaller prostheses. Their smaller cross-sectional area may have contributed to proportionally less resistance to bending stresses. It is possible that these smaller stems were thereby more susceptible to micromotion within the stem compared to midsize and larger stems. With a shorter length, the stem is also less resistant to pivoting around the calcar or midstem.

We now use at least a Corail size 12 stem in patients over 200 pounds to increase the prosthetic stiffness or choose an alternative stem design, even though we acknowledge that the decision is only theoretically based without a control group for study. Further, higher levels of activity are known to correlate with small stem failure [19], and all three patients in this series returned to high levels of activity in the first months. We now modify our postoperative recovery protocol in these large patients with restricted activity for eight weeks.

We speculate that undersizing the stem in relation to the length of the moment arm may also have contributed to failure. In a comparison of standard and lateralized versions of the Corail stem, Cantin et al. noted an incidence of failure in 1.8% of offset stems versus 0% in standard stems [20]. He hypothesized that “lateralized stems are associated with high values of varus and torque moments, which may promote loosening” [20]. We too speculate that this particular mechanical change to the moment arm may affect the stability of small stems in large males, although the NJR data did not differentiate between extended-offset and standard-offset Corail stems in their risk analysis. Mechanically, the offset distance from the center of the femoral head to the axis of the stem body is increased by neck geometry and head length. Unlike other popular stem designs, the Corail maintains a constant neck length between sizes: the smallest size 8 stem has the same neck length and offset as the largest size 20 stem. These larger sizes are not only wider, but also lengthen proportionally to increase the overall potential volume of fixation. The three failed stems in this series had a high offset or coxa vara design, both of which increased offset by an additional 7 mm compared to a standard neck geometry. Further increases in offset occurred with the use of extended head lengths of +5 to +9 mm. Currently, in an effort to minimize the moment arm, we do not use extended head lengths (greater than 1.5 mm) on extended-offset geometries in smaller-size stems. A reduction in offset is considered an acceptable compromise in these situations, as suggested by Cantin [20].

A limitation of this case series is the exclusion of periprosthetic fractures as a significant failure mechanism for small stems in large men. Theoretically, undersized stems prematurely potted distally without metaphyseal engagement can fail into varus and can aseptically loosen or can create a medial calcar fracture as the stem fails into varus. While we have not encountered this mechanism of periprosthetric fracture, we suggest a future study to review periprosthetic fractures with the same patient risk factors and small stems.

## Conclusions

In summary, our three cases of aseptic stem loosening shared the characteristics of being active men with Dorr A bone morphology, weight above 200 pounds, and smaller stem sizes with extended offset. Although axial and rotational stability was achieved at the time of surgery, the perceived stability of the broach in the canal was likely due to distal fixation in the tight canal rather than metaphyseal support. The lack of sufficient metaphyseal support over time led to cantilever bending of the proximal body, as seen by the progression of RLLs in Gruen zones 1 and 7. With excessive loads from high activity, elevated BMIs, and an increased lever arm from extended offset, the smaller stems were unable to fix proximally with bone ingrowth, and shifted into varus.

Ironically, the technique guideline states that cortical contact should be avoided and that the smaller of two sizes should be chosen if in between sizes. Technically, undersizing is a preferred and proven technique according to registry data. We too have found success following these guidelines, but for these rare aseptic failures. These failures, however, have guided us over the past decade to make technical changes to minimize future risks of aseptic loosening. Larger stems, reaming the distal canal, avoiding longer heads with offset stem geometries, and limited postoperative activity, especially in those with elevated BMIs, are now part of our routine. As this study is primarily an examination of past failures, however, the improved efficacy of current techniques is only theoretical. Future studies are warranted to evaluate the efficacy of these changes.
